# Hypoxia upregulates neutrophil degranulation and potential for tissue injury

**DOI:** 10.1136/thoraxjnl-2015-207604

**Published:** 2016-08-31

**Authors:** Kim Hoenderdos, Katharine M Lodge, Robert A Hirst, Cheng Chen, Stefano G C Palazzo, Annette Emerenciana, Charlotte Summers, Adri Angyal, Linsey Porter, Jatinder K Juss, Christopher O'Callaghan, Edwin R Chilvers, Alison M Condliffe

**Affiliations:** 1 Department of Medicine, University of Cambridge, Cambridge, UK; 2 Department of Infection, Immunity and Inflammation, University of Leicester, Leicester, UK; 3 Department of Infection, Immunity and Cardiovascular Disease, University of Sheffield, Sheffield, UK; 4 Department of Respiratory Medicine, Portex Unit, Institute of Child Health, University College London, Cambridge, UK

**Keywords:** Neutrophil Biology, Airway Epithelium, COPD ÀÜ Mechanisms, Innate Immunity, Cystic Fibrosis, Respiratory Infection

## Abstract

**Background:**

The inflamed bronchial mucosal surface is a profoundly hypoxic environment. Neutrophilic airway inflammation and neutrophil-derived proteases have been linked to disease progression in conditions such as COPD and cystic fibrosis, but the effects of hypoxia on potentially harmful neutrophil functional responses such as degranulation are unknown.

**Methods and results:**

Following exposure to hypoxia (0.8% oxygen, 3 kPa for 4 h), neutrophils stimulated with inflammatory agonists (granulocyte-macrophage colony stimulating factor or platelet-activating factor and formylated peptide) displayed a markedly augmented (twofold to sixfold) release of azurophilic (neutrophil elastase, myeloperoxidase), specific (lactoferrin) and gelatinase (matrix metalloproteinase-9) granule contents. Neutrophil supernatants derived under hypoxic but not normoxic conditions induced extensive airway epithelial cell detachment and death, which was prevented by coincubation with the antiprotease α-1 antitrypsin; both normoxic and hypoxic supernatants impaired ciliary function. Surprisingly, the hypoxic upregulation of neutrophil degranulation was not dependent on hypoxia-inducible factor (HIF), nor was it fully reversed by inhibition of phospholipase C signalling. Hypoxia augmented the resting and cytokine-stimulated phosphorylation of AKT, and inhibition of phosphoinositide 3-kinase (PI3K)γ (but not other PI3K isoforms) prevented the hypoxic upregulation of neutrophil elastase release.

**Conclusion:**

Hypoxia augments neutrophil degranulation and confers enhanced potential for damage to respiratory airway epithelial cells in a HIF-independent but PI3Kγ-dependent fashion.

**Key messages**

**What is the key question?**

- Does hypoxia promote neutrophil degranulation and promote tissue injury?

**What is the bottom line?**

- Levels of hypoxia relevant to airway inflammation in vivo markedly increase the release of all neutrophil granule subtypes, augmenting the capacity for neutrophil-mediated bronchial epithelial damage in a phosphoinositide 3-kinase (PI3K)-dependent manner.

**Why read on?**

- We present evidence that tissue hypoxia augments neutrophil-mediated injury, a process that is relevant to airway and lung parenchymal inflammation/infection and amenable to PI3Kγ inhibition.

## Introduction

The neutrophil is the principal cellular effector of innate immune responses and the host's front-line defence against pathogens at mucosal surfaces. Severe neutropenia or defects of neutrophil function confer enhanced susceptibility to infection, and such episodes may be life-threatening. The ‘flip’ side of the neutrophils' protective role is that inappropriate or excessive activation is associated with inflammatory disease, with the powerful neutrophil armamentarium being deployed against delicate host tissues. There is compelling evidence from humans and from experimental animal models that neutrophil influx incites pathological inflammation in multiple organs^1^ and contributes to the pathogenesis of conditions such as acute lung injury,^2^ inflammatory bowel disease^3^ and rheumatoid arthritis.^4^ The propensity of neutrophils to injure surrounding tissues is intimately related to their activation status, with priming for enhanced degranulation and respiratory burst activity a prerequisite for significant damage.^5^

Even in health, neutrophils are exposed to major changes in oxygen availability as they transit between the arterial and venous circulations. In normal tissues, cells located away from capillaries beyond the oxygen diffusion limit (80–140 μm)^6^ are exposed to low oxygen tensions, and hence exist in a state of ‘physiological hypoxia’. Injurious processes such as inflammation and infection amplify physiological hypoxia and comprise ‘pathological hypoxia’. For example, despite direct exposure to atmospheric oxygen, the keratinocyte layer of the skin is a naturally hypoxic milieu^7^; in association with neutrophilic infiltration, the microenvironment of the skin becomes profoundly hypoxic.^8^ Infiltrating neutrophils may contribute directly to oxygen depletion at inflamed surfaces by means of intense use of oxygen by the nicotinamide adenine dinucleotide phophate-oxidase (NADPH oxidase), elegantly demonstrated in the colonic mucosa.^9^

Like the skin, the airway is ‘bathed’ in ambient oxygen, yet increased expression of hypoxia-inducible factor (HIF)-1α has been detected in the bronchial epithelium in COPD.^10^ More compellingly (since inflammatory mediators also upregulate the expression of HIF),^11^ the airway epithelium of mice overexpressing the β-epithelial Na^+^ channel (a model of cystic fibrosis (CF)) stained strongly with the specific hypoxia probe pimonidazole hydrochloride (Hypoxyprobe,^12^ which binds at a threshold of ≤1.3 kPa O_2_^13^), confirming that the inflamed airway is a profoundly hypoxic environment. Neutrophils infiltrate the hypoxic bronchial tissues in patients with COPD,^14^ and airway neutrophilia correlates with decline in lung function^15^ and with HRCT indicators of peripheral airway dysfunction.^16^ Likewise, neutrophilic airway inflammation correlates with disease progression in CF,^17^ and has been linked to hypoxic airway epithelial necrosis.^18^ Finally, respiratory infection is associated with profound local hypoxia, demonstrated directly in animal models by the local uptake of Hypoxyprobe.^19^ Therefore, neutrophils recruited to inflammatory or infective environments at airway mucosal surfaces or within the lung parenchymal tissue are required to function under conditions of significant hypoxia.

Neutrophils are adapted to function in hypoxic environments, and even under conditions of high oxygen tension, they rely predominantly on glycolysis for ATP generation.^20^ While the effector functions of these cells have been studied extensively under conditions of ambient oxygen, the few studies that have examined the ability of these cells to operate under conditions of hypoxia have demonstrated profound effects on both function and longevity. We have previously shown that hypoxia enhances neutrophil survival by inhibition of apoptosis via HIF-1α-mediated nuclear factor (NF)-κβ activation.^21^ More recent data from our laboratory has shown that while neutrophil chemotaxis and phagocytosis are preserved under conditions of profound hypoxia, the ability to mount an oxidative burst and consequent killing of organisms such as *Staphylococcus aureus* are markedly impaired.^22^

Since neutrophil-derived proteases play such a key role in the pathogenesis of COPD^23^ and CF,^24^ and local tissue hypoxia is highly relevant to airway inflammation in these conditions, we explored the impact of hypoxia on neutrophil degranulation responses. We found that hypoxia markedly upregulates the release of all major neutrophil granule populations, to an extent that promotes damage to respiratory epithelial cells in culture. This hypoxic potentiation of degranulation and injurious potential was not mediated by HIF-1α, a pivotal regulator of hypoxic signalling, but was associated with enhanced basal and cytokine-stimulated neutrophil AKT phosphorylation. Inhibitor studies demonstrated a role for phosphoinositide 3-kinase (PI3K)γ in mediating hypoxia-enhanced degranulation.

## Methods

### Isolation and culture of peripheral blood neutrophils

Cambridge Research Ethics Committee granted ethical approval (06/Q0108/281). Neutrophils were purified from healthy volunteers by dextran sedimentation and discontinuous plasma–Percoll gradients.^25^ Purified (>98% pure) cells were resuspended at 5–11×10^6^/mL in Iscove's Modified Dulbecco's Medium (IMDM). Hypoxia was established using an InvivO_2_ 400 hypoxic workstation (Ruskinn, Bridgend, UK), with pre-equilibration of media. Normoxic incubations used IMDM equilibrated under ambient atmospheric conditions at 37°C. The pO_2_, pCO_2_ and pH of the media were measured (ABL80 Blood Gas Analyzer; Radiometer, Copenhagen, Denmark) at the beginning and at the end of each experiment (see refs. 21, 22 and supplementary figure S1A).

10.1136/thoraxjnl-2015-207604.supp1 supplementary figures

### Determination of neutrophil degranulation

Neutrophils (3×10^6^ in 270 μL medium) were incubated in IMDM under normoxia or hypoxia for 4 h. Granulocyte-macrophage colony stimulating factor (GM-CSF) (10 ng/mL), tumour necrosis factor-alpha (TNFα) (20 ng/mL), platelet-activating factor (PAF) (1 μM) or vehicle control were added for 5–30 min as indicated, followed by the formylated peptide (fMLP) (100 nM) or vehicle for 10 min. After centrifugation, degranulation was assessed by measuring elastase, myeloperoxidase (MPO), lactoferrin and matrix metalloproteinase-9 (MMP-9) in the supernatants. Active elastase release was measured using a commercial kit (EnzChek, Life Technologies, UK). MPO secretion was measured by the H_2_O_2_-dependent oxidation of o-Dianisidine dihydrochloride.^26^ Lactoferrin (Hycult Biotech, Uden, The Netherlands) and MMP-9 (R&D, UK) release were measured using commercial ELISA', according to the manufacturers’ instructions. MMP-9 activity was quantified by gelatine zymography.^27^

### Neutrophil apoptosis and neutrophil extracellular trap formation

Neutrophils were cytocentrifuged and stained with May-Grünwald–Giemsa (Merck); apoptotic neutrophils were defined by pyknotic nuclei. Fluorescein isothiocyanate (FITC)-annexin–TOPRO-3 staining (Invitrogen) was undertaken according to manufacturers’ instructions. For NETosis, neutrophils (10^6^/mL) were incubated with SYTOX Green (1:1000), and after 4 h treated with GM-CSF and formylated peptide (fMLP). Extracellular DNA was measured by fluorescence absorbance at 485/535 nm, and NETosis was calculated as percentage of total DNA from cells lysed at baseline.

### Small molecule inhibitors

The pan-PI3K inhibitor LY294002 (10 µM), PI3Kγ inhibitor AS605240 (3 µM), PI3Kδ inhibitor IC87114 (3 µM), cycloheximide (1 µ/mL), thapsigargin (100 nM) and phospholipase C (PLC) inhibitor U-73122 (2 µM) were purchased from Sigma. Jasplakinolide (500 nM) was purchased from Tocris Bioscience. U-73122 was added 10 min prior to stimulation (fMLP), jasplakinolide 5 min prior to stimulation and thapsigargin immediately prior to stimulation. PI3K inhibitors were added to cells either at the beginning of the normoxic/hypoxic incubation or 10 min before fMLP stimulation. Cycloheximide and ethylene glycol tetraacetic acid (EGTA) (2 mM) were also present from the outset of the normoxic/hypoxic incubation.

### Preparation of cigarette smoke extract

Cigarette smoke extract (CSE) was made by bubbling smoke from three research-grade 3R4F cigarettes (Kentucky Tobacco Research Institute) through 25 mL of IMDM. This solution was designated as 100% CSE and diluted as indicated.

### Epithelial cell culture

A549 cells (American Type Culture Collection (ATCC), passage 2–8) were cultured in F-12K medium with penicillin (100 U/mL), streptomycin (100 µg/mL), amphotericin B (25 µg/mL) and 10% fetal calf serum. Immortalised human bronchial epithelial cells^28^ (iHBECs) were cultured in keratinocyte serum-free media supplemented with bovine pituitary extract (25 μg/mL), recombinant epidermal growth factor (rEGF) (0.2 ng/mL), puromycin (250 ng/mL) and G418 (25 μg/mL, Fisher Scientific, Loughborough, UK). Primary normal human bronchial epithelial cells (NHBE) cells (Lonza, UK) were grown to a ciliated phenotype at air–liquid interface.

### 3-(4,5-dimethylthiazol-2-yl)-2,5-diphenyltetrazolium Bromide (MTT) assay

Confluent A549 cell layers were exposed to neutrophil supernatants or vehicle controls for 72 h at 37°C. Following incubation with MTT (500 µg/mL, 2 h, 37°C), cells were dissolved in 100 µL isopropanol, and absorbance at 540 nm was recorded.

### Immunohistochemistry and electron microscopy of epithelial cell layers

Confluent A549/iHBEC cell layers were exposed to neutrophil supernatants for 48 h, fixed with 4% paraformaldehyde, stained for F-actin with rhodamine phalloidin (Invitrogen; 1:200, 30 min) and mounted in ProLong Gold Antifade Mountant (Invitrogen, UK), with 4',6-diamidino-2-phenylindole (DAPI) as the nuclear stain. Quantification (imageJ) measured the area of cell detachment, three fields of view per well, three wells per condition. For electron microscopy, NHBE cells were fixed in 4% glutaraldehyde containing 0.1% (v/v) hydrogen peroxide and osmium tetroxide (2 h at 4°C), embedded in Spurr’'s resin and imaged using a Philips CM 100 transmission electron microscope (TEM).

### Measurement of ciliary beat frequency

To determine ciliary beat frequency (CBF), ciliated NHBE cells were exposed to neutrophil supernatants prior to transfer to a chamber slide. Beating cilia were recorded using a Motion Pro X4 digital high-speed video camera (Lake Image Systems, Henrietta, New York, USA) at 250–500 frames/s using a 40× objective as previously described.^29^ A minimum of 512 or 1024 frames were captured, respectively. Video sequences were analysed at reduced frame rates and CBF calculated using ciliaFA.^30^

### TaqMan real-time PCR and western blotting

RNA was isolated using TRI reagent (Sigma-Aldrich, Gillingham, UK) followed by RNA purification with DNase digest (RNeasy micro columns, Qiagen, Manchester, UK). cDNA was generated from 1 μg total RNA, using a High-Capacity cDNA Kit (Applied Biosystems, Foster City, California, USA), and quantitative PCR (qPCR) (iCycler; Bio-Rad, Milpitas, California, USA) was performed using SYBR Green Master Mix (Sigma-Aldrich) and commercial primers (Qiagen). Relative gene expression was determined by correcting cycle thresholds for target genes against housekeeping genes (β-actin, tyrosine 3-monooxygenase/tryptophan 5-monooxygenase activation protein zeta (YWHAZ)) using GenNorm (http://medgen.ugent.be/~jvdesomp/genorm). The ΔCT for the target gene of interest in control, normoxic-stimulated and hypoxic-stimulated neutrophils was corrected against the value obtained in freshly isolated neutrophils to give ΔΔCT values. Fold-change in gene expression is expressed as 2^−ΔΔCT^. Western blotting was performed using rabbit polyclonal anti-MMP-9 (Abcam), rabbit polyclonal AKT and rabbit monoclonal serine 473-phospho-AKT (pAKT) (Cell Signalling Technology).

### Statistical analysis

Data are reported as mean±SEM from (n) independent experiments. Gaussian data were analysed using analysis of variance with Holm–Sidak correction for multiple comparisons, or paired t test where appropriate. Data that could not be assumed to be from a Gaussian population were analysed using Kruskal–Wallis with Dunn's post-test. All analyses were undertaken using Prism 6.0g software (GraphPad, San Diego, California, USA). A p value of <0.05 was considered significant, *p<0.05, **p<0.01, ***p<0.005, ****p<0.001.

## Results

### Hypoxia augments neutrophil degranulation

**Figure 1:**
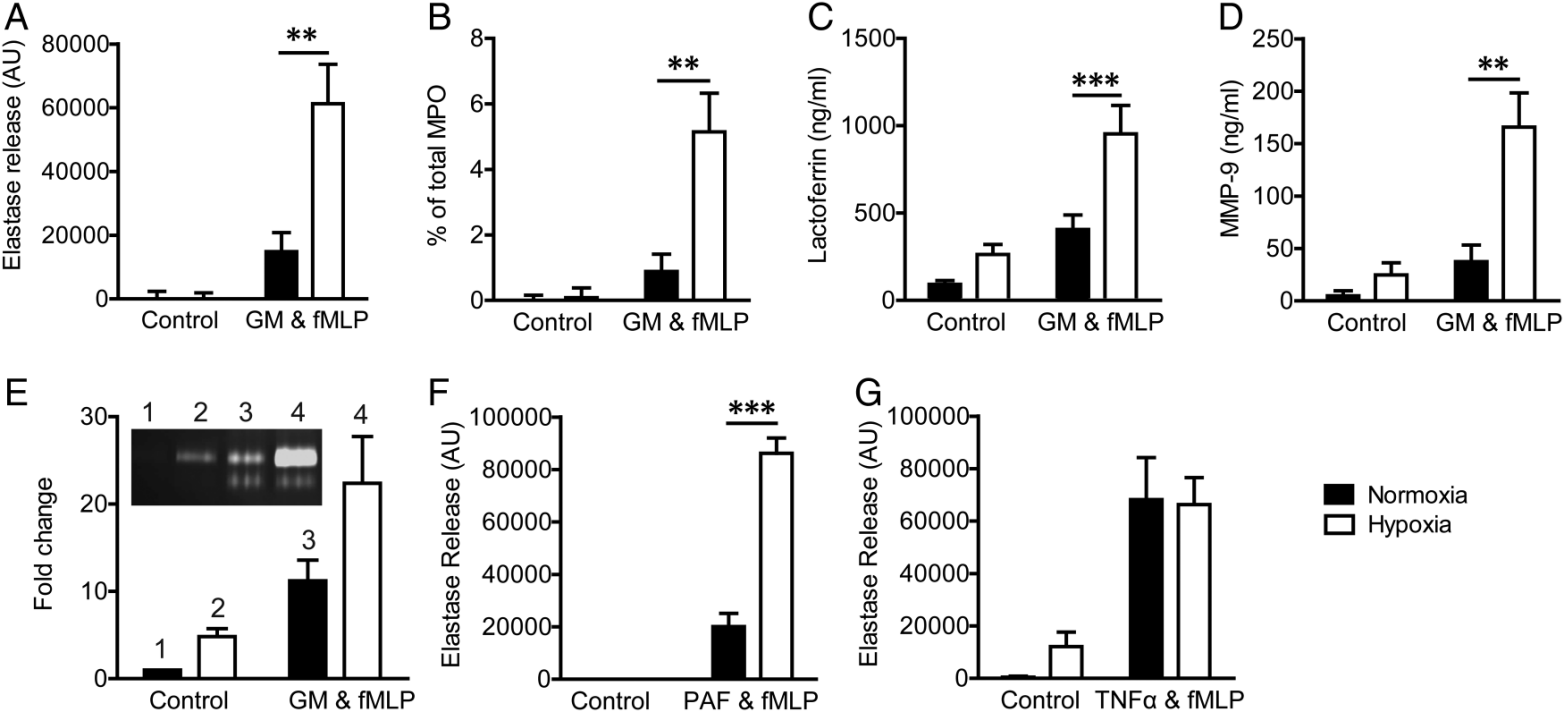
Hypoxia augments neutrophil degranulation. (A–E) Hypoxia augments the release of all granule populations. Neutrophils were incubated under normoxia or hypoxia for 4 h, primed (granulocyte-macrophage colony stimulating factor (GM-CSF); 10 ng/mL, 30 min) or vehicle controlled and activated (formylated peptide (fMLP); 100 nM, 10 min), and the supernatants were assayed for the presence of granule proteins. Hypoxia augmented the release of active elastase (A, n=10, each performed in triplicate) as measured by elastase activity assay, active myeloperoxidase (MPO) (B, n=8, each performed in triplicate) as measured by the H_2_O_2_-dependent oxidation of O-dianisidine dihydrochloride, lactoferrin as measured by ELISA (C, n=6) and active matrix metalloproteinase-9 (MMP-9) as measured by ELISA (D, n=5, each performed in triplicate) and gelatine zymography (E, n=3, each performed in triplicate plus representative zymogram, inset). (F and G) Neutrophils were incubated under normoxia or hypoxia for 4 h prior to priming with platelet-activating factor (PAF) (1 µM, 5 min, n=4, each performed in triplicate) or TNFα (G, 20 ng/mL 30 min, n=10, each performed in triplicate) or media control and activation with fMLP (100 nM, 10 min), and the supernatants were assessed by elastase activity assay as above. Results represent mean±SEM. **p<0.01, ***p<0.005.

Neutrophils cultured in ambient air (‘normoxia’) released minimal active neutrophil elastase (NE) even when stimulated with physiological agonists (GM-CSF and fMLP in combination). Hypoxic incubation (3 kPa, 4 h) increased the stimulated release of active NE up to sixfold (figure 1A). To determine whether this reflected augmented azurophil granule release or was specific to NE alone, MPO activity was also assessed, and an identical pattern of hypoxia-enhanced release was observed (figure 1B). Hypoxia also increased the detection of extracellular lactoferrin (specific granules: figure 1C) and active MMP-9 (gelatinase granules: figure 1E), demonstrating that hypoxia augments the release of all neutrophil granule populations, both basally and on stimulation with physiological agonists. Equivalent increases in total NE,^22^ lactoferrin and MMP9 (figure 1C, D) were detected by ELISA. Hypoxia also upregulated fMLP-stimulated NE secretion from PAF-primed but not TNFα-primed neutrophils (figure 1F, G), demonstrating that this effect is agonist-specific and does not equate to priming, as all three agonists augmented the neutrophil oxidative burst (data not shown). Neutrophil survival under all conditions was confirmed by trypan blue exclusion, microscopy and FITC-annexin staining (see online supplementary figure S1B/C); hence, the augmented degranulation response did not reflect loss of membrane integrity or cell death. Furthermore, hypoxia did not increase neutrophil extracellular trap (NET) release in response to hypoxia alone or in combination with GMCSF/fMLP (see online supplementary figure S1D). We saw no significant effect of CSE on neutrophil degranulation under normoxic or hypoxic conditions (see online supplementary figure S2), although CSE did induce the intracellular generation of oxygen species (ROS), confirming biological activity; the minor uplift of NE release seen under normoxia with 50% CSE reflected reduced neutrophil viability, and both were prevented by hypoxia (data not shown).

### Hypoxia increases the injurious potential of neutrophil supernatants

**Figure 2:**
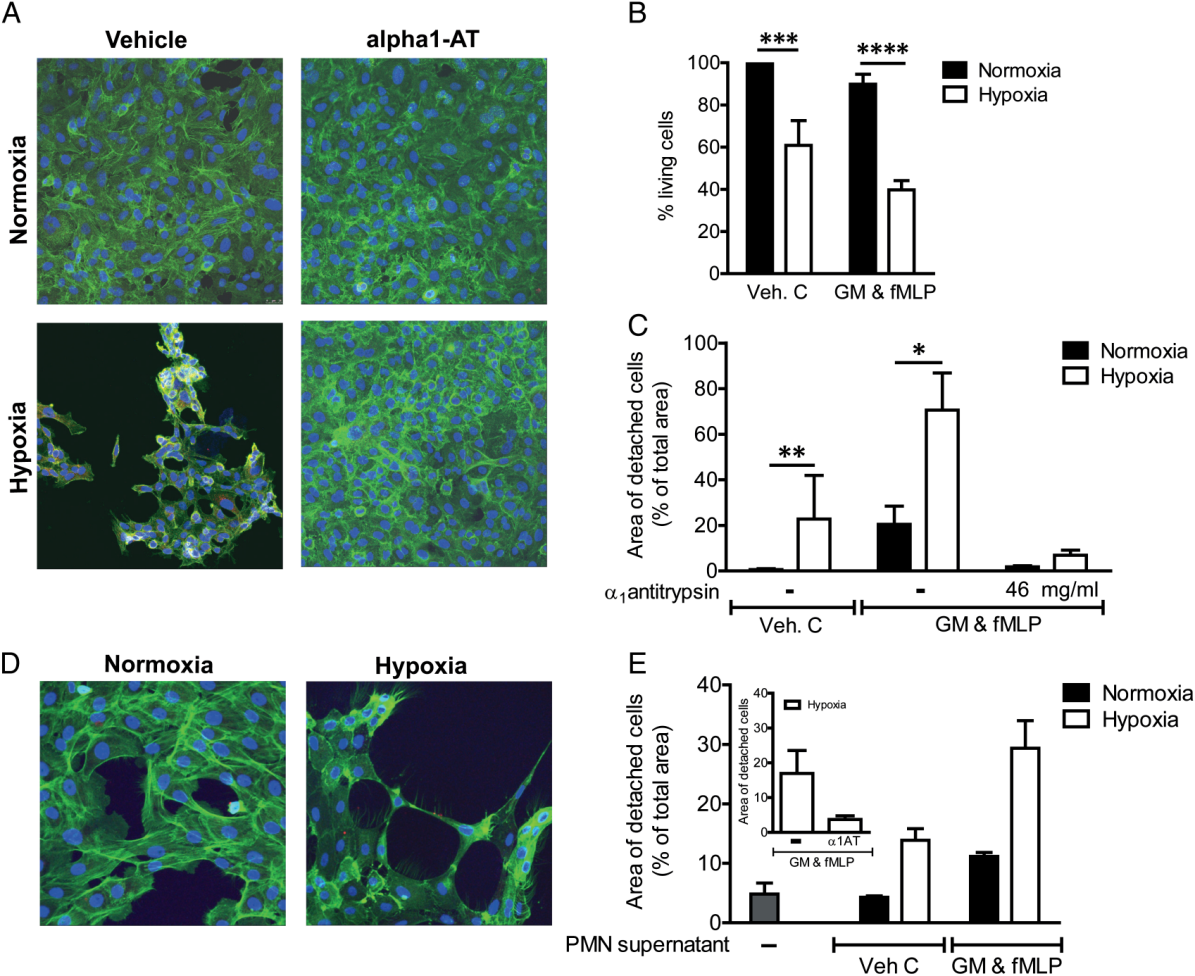
Hypoxia increases neutrophil supernatant-mediated epithelial injury. (A–C) Hypoxia increased the injurious potential of neutrophil supernatants to A549 cells. Confluent cell monolayers were exposed to diluted (1:2.5) supernatants (from neutrophils incubated under normoxic or hypoxic conditions for 4 h, without (control) or with GM-CSF (GM) and formylated peptide (fMLP) activation)±α_1_AT (46 μg/mL) for 48–72 h. At 48 h, cell layers were stained (A, representative images from n=6 experiments, each performed in triplicate) for cleaved caspase 3 (red), F-actin (green) and nuclei (blue). Survival of the A549 cells at 72 h was measured by MTT assay (B, n=11, each performed in triplicate; ***p<0.005, ****p<0.001). Cell layer detachment was quantified with ImageJ (C, n=3, three fields of view per well, performed in triplicate; *p<0.05, **p<0.01 (paired t test)). (D and E) Hypoxia increased the injurious potential of neutrophil supernatants to immortalised human bronchial epithelial cells28 (iHBECs). Confluent cell layers were exposed to neutrophil supernatants (1:2.5 dilution) for 48 h. At 48 h, cell layers were stained (D, representative images from n=3, each performed in triplicate) for cleaved caspase 3 (red), F-actin (green) and nuclei (blue). Cell layer detachment was quantified with ImageJ (E, n=3, each performed in triplicate). Results represent mean±SEM.

**Figure 3:**
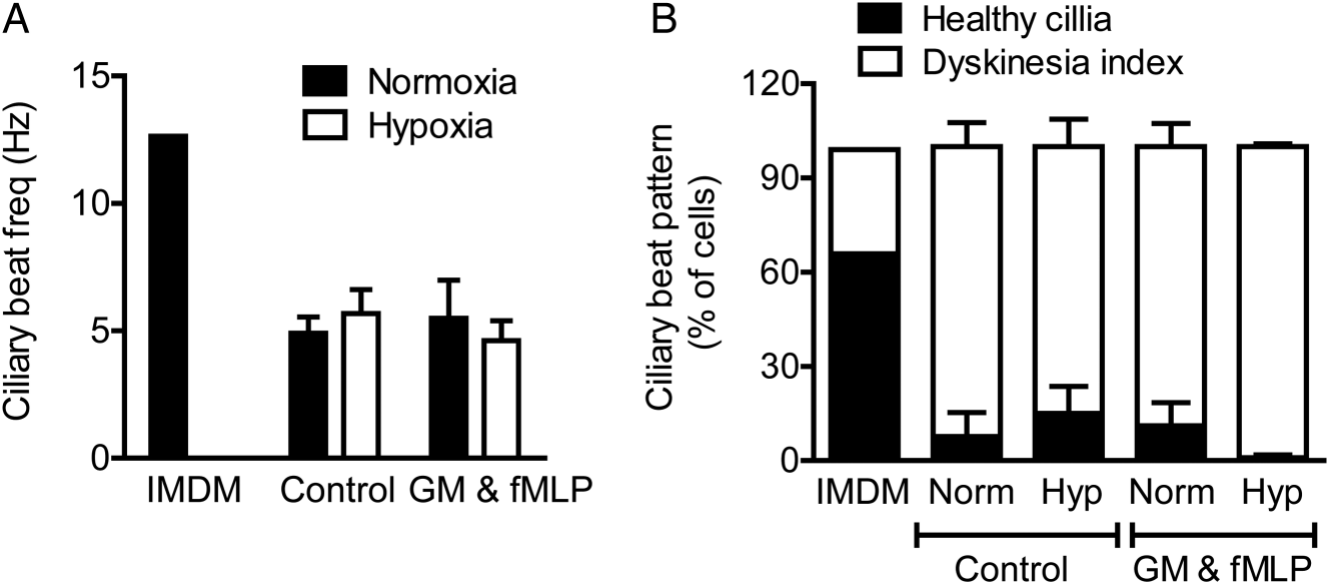
Neutrophil supernatants impair ciliary function. Supernatants from neutrophils incubated under normoxic or hypoxic conditions for 4 h, without (control) or with GM-CSF (GM) and formylated peptide (fMLP) activation were applied to strips of ciliated primary human bronchial epithelial cells for 2 h. Ciliary function was assessed by high-speed digital video microscopy allowing ciliary beat frequency (CBF, A) and detailed ciliary beat pattern (CBP, B) analysis as detailed in the Methods section. Results represent mean±SEM (n=3, three movies per ciliated strip of epithelial cells).

Neutrophil-derived proteases and proteins released on degranulation cause lung tissue damage.^31^ Supernatants from normoxic and hypoxic neutrophils stimulated with GM-CSF and fMLP were therefore applied to cultured A549 cells, and monolayer integrity was assessed. Hypoxic neutrophil supernatants induced substantial epithelial cell detachment and death (figure 2A, B). Coincubation with the endogenous protease inhibitor α-1 antitrypsin protected A549 cells from injury (figure 2A, C), suggesting that neutrophil-derived proteases are responsible for this effect. These experiments were recapitulated using iHBECs, and again hypoxia augmented the injurious potential of the neutrophil supernatants (figure 2D, E), with α-1 antitrypsin conferring protection (figure 2E, inset). Both normoxic and hypoxic supernatants compromised CBF and ciliary coordination when applied to cultured primary NHBE cells (see figure 3A, B and supplementary videos S1 and S2), suggesting that this function is exquisitely sensitive to even minimal exposure to neutrophil granule products, or that an alternative (non-degranulation-dependent) agent in the supernatant is responsible. Hypoxic supernatants induced subtle features of cell damage assessed by electron microscopy at early time points, with a trend to enhanced lactate dehydrogenase (LDH) release at later times (see supplementary figure S3).

10.1136/thoraxjnl-2015-207604.supp2 supplementary video

10.1136/thoraxjnl-2015-207604.supp3 supplementary video

### Hypoxic upregulation of neutrophil degranulation is not HIF-dependent and does not depend on cytoskeletal remodelling

**Figure 4:**
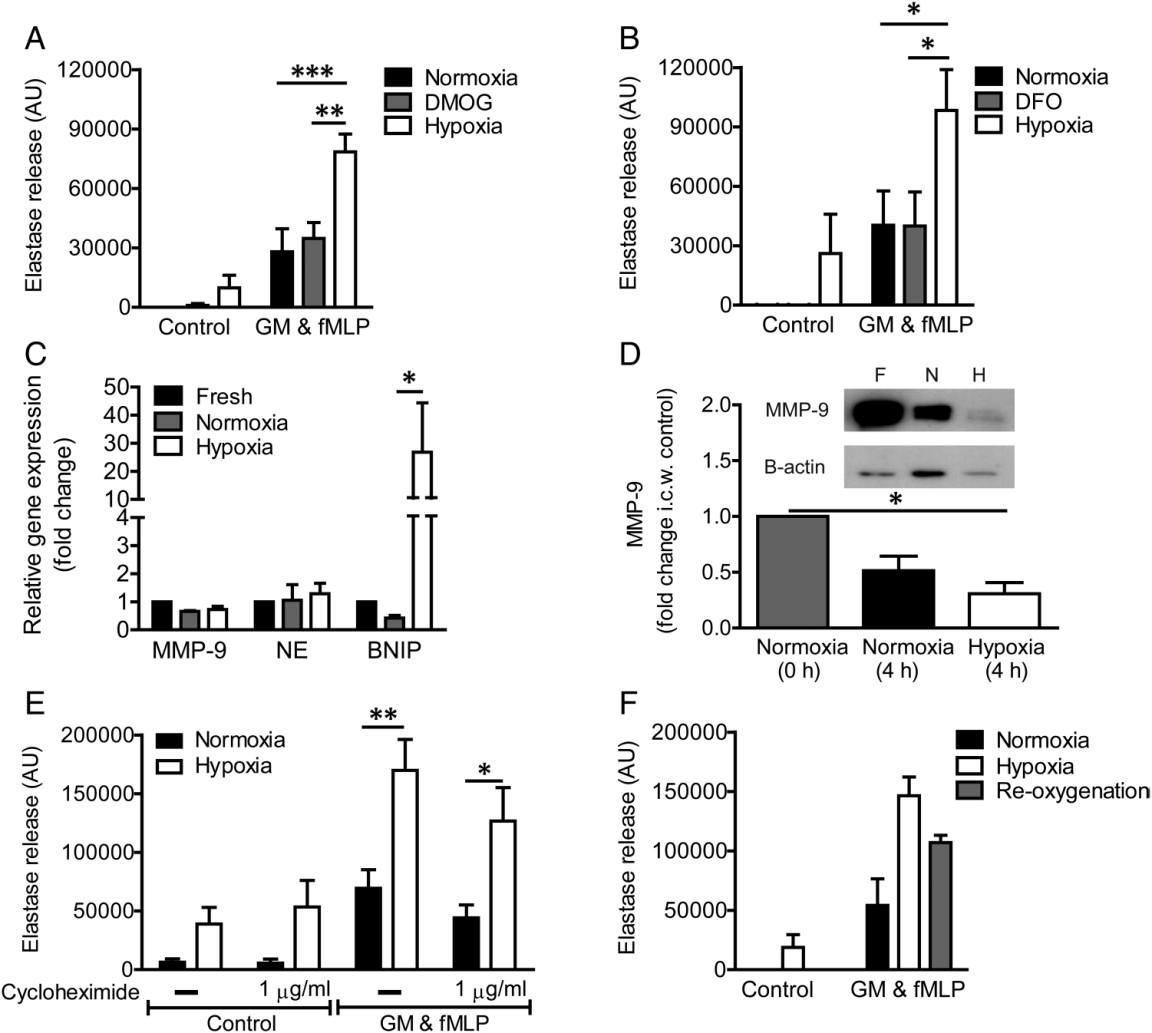
Hypoxic upregulation of neutrophil degranulation is not hypoxia-inducible factor (HIF)-dependent. (A and B) Hypoxia mimetics do not recapitulate the hypoxic upregulation of neutrophil degranulation. Neutrophils were incubated under normoxia for 4 h±DMOG (dimethyloxalyl glycine) (1 mM, A) or desferrioxamine (DFO) (1 mM, B) or were incubated under hypoxia (positive control), prior to priming with GM-CSF (GM) (10 ng/mL, 30 min) and activation with formylated peptide (fMLP) (100 nM, 10 min). Supernatants were assayed for active elastase. Results represent mean±SEM (n=5–6, each performed in triplicate). (C–E) Hypoxia does not increase granule protein transcription, translation or total protein. Neutrophils were incubated under normoxia or hypoxia for 4 h before RNA isolation or protein quantification. (C) Granule protein (NE, neutrophil elastase and MMP-9, matrix metalloproteinase-9) and BNIP (BCL2/adenovirus E1B 19 kd-interacting protein-2, positive control) gene expression as measured by quantitative PCR (qPCR); results represent mean±SEM (n=3, each performed in triplicate). (D) Gelatinase granule content measured by western blot analysis of MMP-9; results represent mean±SEM (n=3). (E) Hypoxic elastase release was not prevented when translation was inhibited by cycloheximide, present for the entire incubation, prior to stimulation with GM-CSF (GM) and fMLP. Results represent mean±SEM (n=5, each performed in triplicate). (F) Reoxygenation (15 min) of neutrophils before priming and activation does not significantly reduce degranulation as measured by the release of active elastase. Results represent mean±SEM (n=5, each performed in triplicate). *p<0.05, **p<0.01, ***p<0.005.

The transcription factor HIF-1α mediates most signalling events initiated by hypoxia; prolongation of neutrophil lifespan under conditions of hypoxia is mediated by the stabilisation of HIF-1α and its interaction with the NF-κβ pathway.^21^ However, two main lines of evidence suggest that HIF-1α is not the principal regulator of the hypoxic upregulation of neutrophil degranulation (figure 4). First, incubation of neutrophils with the hypoxia mimetics dimethyloxalyl glycine (DMOG) and desferrioxamine, which stabilise HIF, did not recapitulate the effects of hypoxia on the release of NE (figure 4A, B) or other granule products (not shown). Second, hypoxic incubation did not increase the neutrophil content of the relevant granule proteins, either at the message (figure 4C) or protein level (figure 4D); indeed, hypoxia accelerated the time-dependent loss of MMP-9 protein, perhaps in keeping with the basal secretion that occurs even in the absence of agonist stimulation on hypoxic incubation (figure 1D). The enhanced transcription of the known HIF target BNIP-2 (BCL2/adenovirus E1B 19 kd-interacting protein-2, figure 4C) confirms the engagement of the HIF pathway under these experimental conditions. Furthermore, incubation with cycloheximide had no effect on the degranulation response observed under either normoxic or hypoxic conditions (figure 4E). In addition, brief reoxygenation of hypoxic cells (which restores the impaired oxidative and killing capacity)^22^ did not affect NE release, suggesting that this effect is not a consequence of altered ROS-dependent signalling (figure 4F).

**Figure 5:**
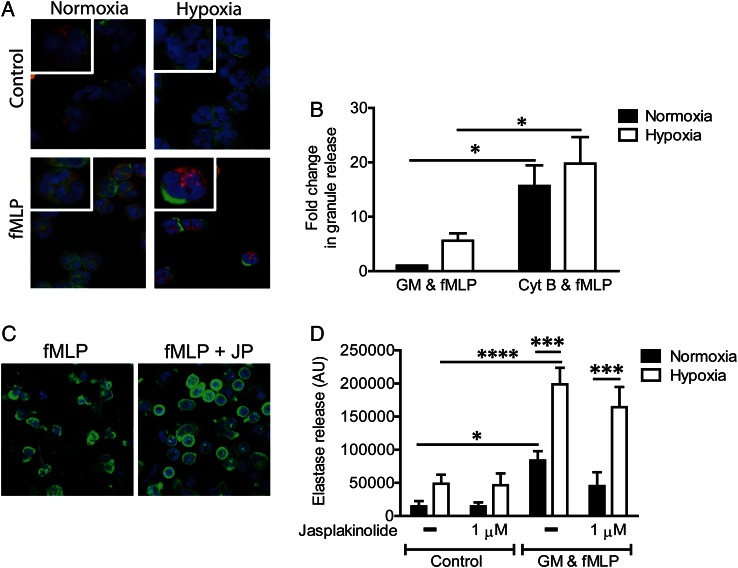
Hypoxic upregulation of neutrophil degranulation does not depend on cytoskeletal remodelling. (A) Neutrophils were incubated under normoxia or hypoxia for 4 h to activation with formylated peptide (fMLP) (100 nM, 5 min). Cells were stained for F-actin (green), neutrophil elastase (red) and nuclei (blue). Representative images are from n=3 experiments, each performed in triplicate. (B) Neutrophils were incubated under normoxia or hypoxia for 4 h prior to priming with cytochalasin B (Cyt B: 5 μg/mL, 5 min) and activation with fMLP (100 nM, 10 min); degranulation was measured by the release of active elastase. Results represent mean±SEM (n=10, each performed in triplicate). (C and D) Induction of actin polymerisation induces the formation of actin ‘rings’, but does not affect neutrophil degranulation. (C) Neutrophils incubated with jasplakinolide (1 μM, 5 min) prior to activation with fMLP (100 nM, 10 min). Cells were stained for F-actin (green) and nuclei (blue). (D) Neutrophils were incubated under normoxia or hypoxia for 4 h prior to priming with GM-CSF (GM) (10 ng/mL, 30 min), treatment with jasplakinolide (1 μM, 5 min) prior to activation with fMLP (100 nM, 10 min). Degranulation was measured by the release of active elastase. Results represent mean±SEM (n=4, each performed in triplicate). *p<0.05, ***p<0.005, ****p<0.001.

Since the actin cytoskeleton has been implicated in degranulation responses, we explored the role of actin polymerisation under hypoxia. Hypoxic incubation promotes focal neutrophil actin polymerisation, leading to the formation of ‘actin caps’ (figure 5A). Cytochalasin B inhibits actin polymerisation and primes neutrophils for NE release more potently than does GM-CSF (figure 5B), but jasplakinolide (which promotes actin polymerisation and induces the formation of subcortical actin ‘rings’, figure 5C) did not diminish the hypoxic upregulation of NE release (figure 5D). Thus, although hypoxia can modulate actin polymerisation,^32^ this mechanism does not underpin the enhanced degranulation we observed.

### PI3K and PLC signalling pathways contribute to the regulation of neutrophil degranulation

**Figure 6:**
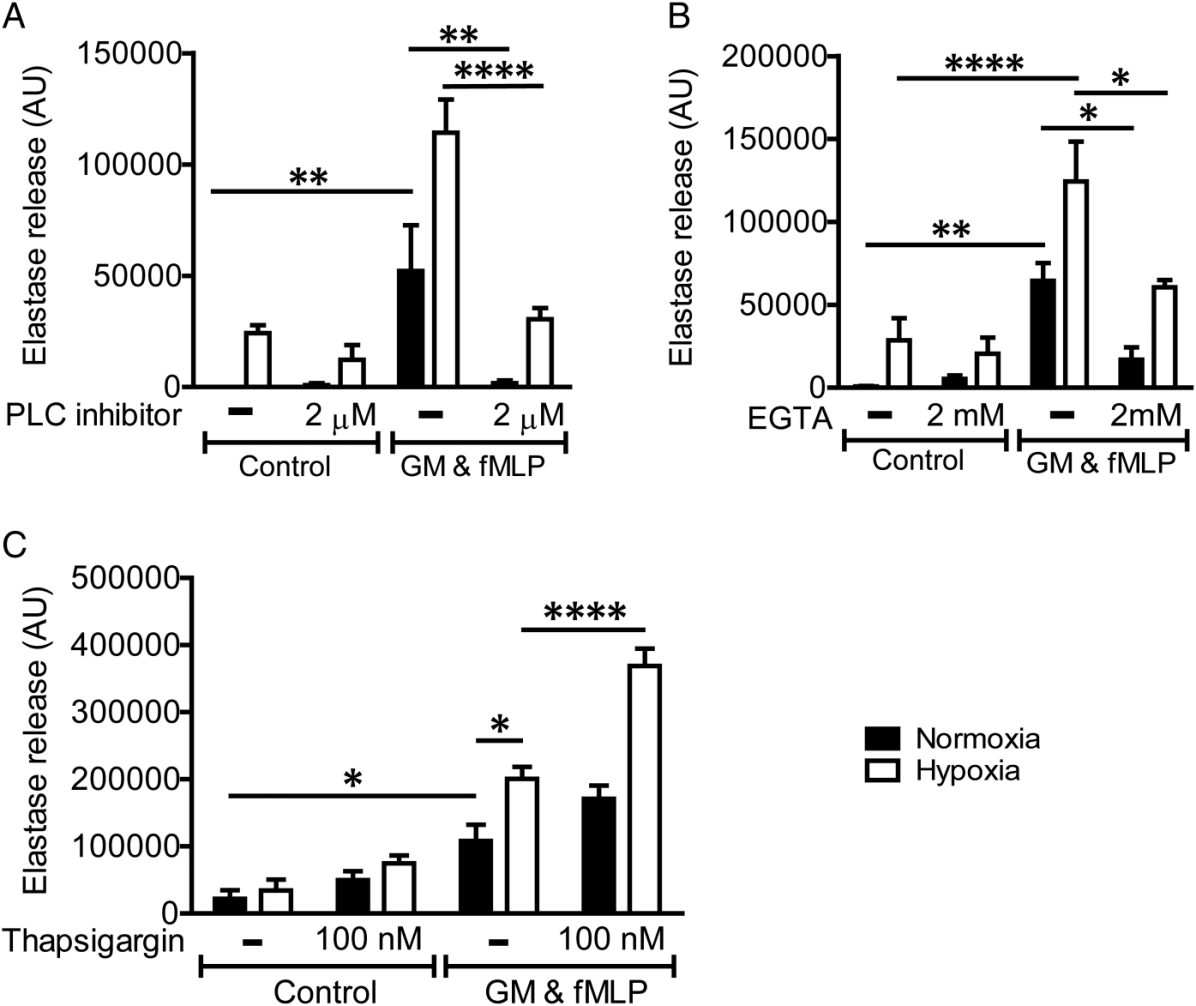
Phospholipase C (PLC) and calcium signalling regulate neutrophil degranulation but do not mediate the hypoxic uplift. Inhibition of the PLC pathway (A) or calcium signalling (B) abolished neutrophil elastase release induced by agonist stimulation under normoxia, and reduced but did not abolish degranulation under conditions of hypoxia. Increasing intracellular calcium (C) induced an increase in degranulation, but similarly did no abolish the hypoxic uplift induced by hypoxia. Neutrophils were incubated under normoxia or hypoxia for 4 h prior to priming with GM-CSF (GM)) (10 ng/mL, 30 min). Neutrophils were preincubated with a PLC inhibitor (U-73122, 2 μM) for 10 min prior to activation with formylated peptide (fMLP) (100 nM, 10 min). EGTA (2 mM) was added at the start of the normoxic/hypoxic incubation, and thapsigargin (100 nM) was added immediately prior to neutrophil activation with fMLP. Cells were spun down and degranulation measured by the release of active elastase. Results represent mean±SEM (n=4–5, each performed in triplicate). *p<0.05, **p<0.01, ****p<0.001.

Since the hypoxic augmentation of neutrophil degranulation was independent of HIF, the so-called ‘master-regulator’ of hypoxic signalling, we explored the roles of the PI3K and PLC/Ca^2+^ signalling pathways, which are also known to contribute to the degranulation response. Inhibition of the PLC pathway (figure 6A) and also chelation of extracellular calcium with EGTA (figure 6B) abolished NE release induced by agonist stimulation under normoxia, and reduced but did not abolish degranulation under conditions of hypoxia; thus ‘the hypoxic uplift’ of this response was still apparent (figure 6B). A non-significant trend to increased elastase release was seen on treatment with thapsigargin (figure 6C) to augment intracellular calcium release, again with preservation of the hypoxic effect.

**Figure 7:**
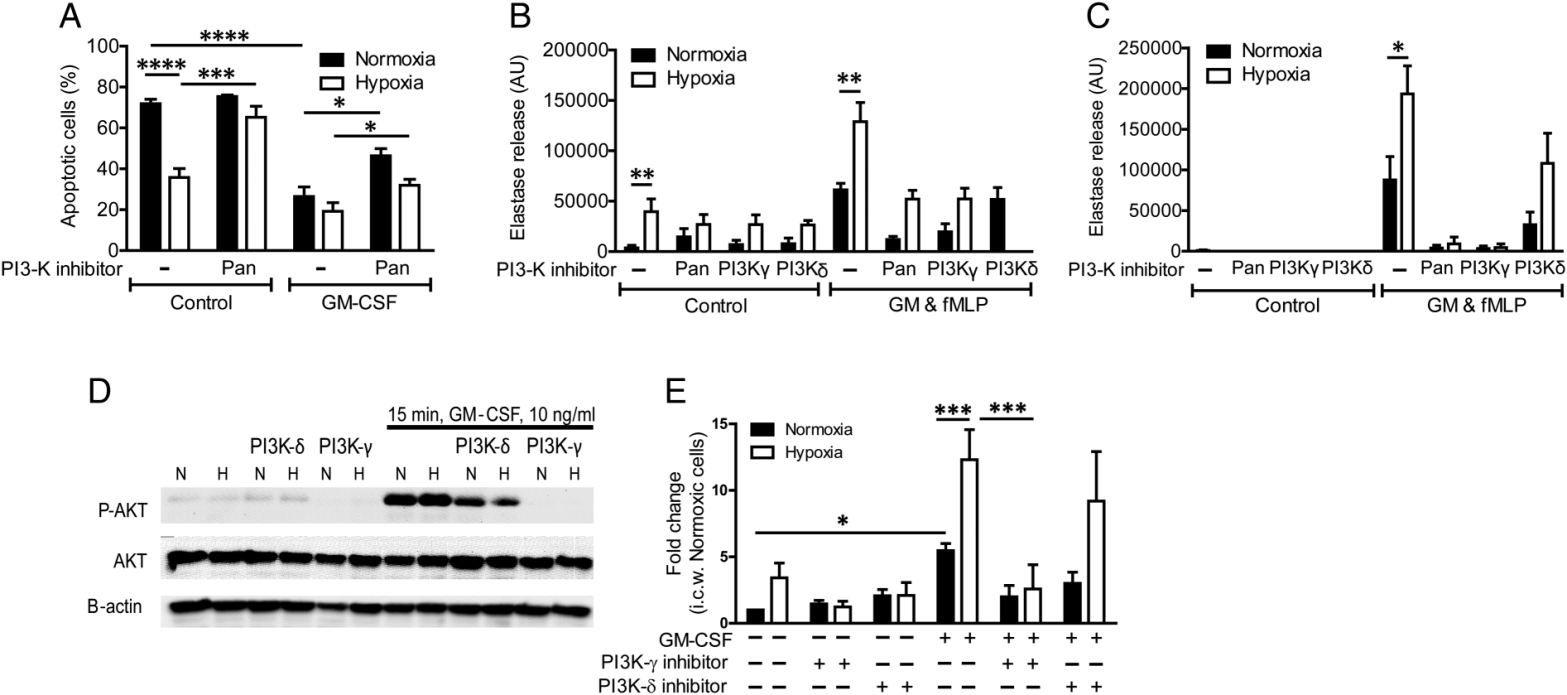
Phosphoinositide 3-kinase (PI3K) signalling contributes to the hypoxic uplift of neutrophil degranulation. (A) Hypoxia induces neutrophil survival at 20 h, which is prevented by incubation with the pan-PI3K inhibitor LY294002 (10 μM). Results represent mean±SEM (n=4, each performed in triplicate). (B and C) Inhibition of the PI3K pathway inhibits degranulation and abrogates the hypoxic response. Neutrophils were incubated under normoxia or hypoxia for 4 h prior to priming with GM-CSF (GM) (10 ng/mL, 30 min). Cells were treated with a pan-PI3K inhibitor (LY294002, 10 μM), a PI3Kγ-selective inhibitor (AS605240, 3 μM), a PI3Kδ-selective inhibitor (IC87114, 3 μM), either for 10 min prior to activation with formylated peptide (fMLP) (B) or from the outset of the normoxic/hypoxic incubation (C). Degranulation was measured by the release of active elastase. Results represent mean±SEM (n=5). (D and E) Hypoxia increases AKT serine-473 phosphorylation after 15 min of GM-CSF stimulation in a PI3Kγ-dependent fashion. Neutrophils were incubated under normoxia or hypoxia for 4 h prior to priming with GM-CSF (10 ng/mL, 15 min). Isoform-selective PI3K inhibitors AS605240 (3 μM, PI3Kγ) and IC87114 (3 μM, PI3Kδ) were added at the start of the incubation. p-AKT was quantified by western blotting. Results represent mean±SEM (n=4–11). *p<0.05, **p<0.01.

Hypoxia prolonged neutrophil survival, an effect that was somewhat diminished by pretreatment with LY294002 (figure 7A), suggesting that hypoxia might entrain the PI3K signalling pathway. Application of a pan-PI3K inhibitor prior to fMLP stimulation abolished normoxic but not hypoxic degranulation responses (figure 7B); however, when the inhibitor was added from the outset of the hypoxic incubation, the hypoxic uplift was completely abrogated and NE release was effectively abolished (figure 7C). In keeping with a possible role for PI3K in mediating the hypoxic upregulation of neutrophil degranulation, AKT phosphorylation in response to GM-CSF was elevated with hypoxic incubation, and occurred in an accelerated fashion (figure 7D, E). In contrast, AKT phosphorylation following fMLP treatment was not modulated by hypoxia (see online supplementary figure S4), suggesting that neutrophil hyper-responsiveness is entrained in response to earlier signalling events. Notably, the hypoxic enhancement of basal and peak GM–CSF-stimulated AKT phosphorylation was completely reversed by inhibition of PI3Kγ (figure 7E).

## Discussion

Neutrophils are generated within a hypoxic bone marrow niche, are exposed to intermittent hypoxia in the circulation and are recruited to the sites of infection and inflammation, which are almost invariably hypoxic. Despite this, most knowledge of neutrophil signalling and function derives from studies conducted under atmospheric oxygen tensions, not reflecting the in vivo physiological or pathological setting. We demonstrate that hypoxic culture (at levels relevant to human disease in vivo) augments neutrophil degranulation, conferring an enhanced injurious potential that may be relevant to neutrophilic airway inflammation in diseases such as COPD and CF, and to inflammatory responses in the wider setting.

We show by ELISA that hypoxia increases the liberation of the cognate proteins from specific (lactoferrin) and gelatinase (MMP-9) granule populations, consistent with our previous report of enhanced release of NE from azuophilic granules.^22^ Importantly we demonstrate herein that NE, MPO and MMP-9 are released by hypoxia in a bioactive form, suggesting that concomitant release of neutrophil antiproteases such as α-1 antitrypsin^33^ is insufficient to neutralise the liberated protease activity. However, exogenous α-1 antitrypsin protected airway epithelial cells from damage induced by hypoxic neutrophil supernatants, confirming the key role of granule serine proteases in mediating this cellular injury. While endogenous antiproteases may be present in inflamed sites, such local defences fail to protect target cells in the immediate vicinity of a degranulating neutrophil, in part due to the phenomenon of quantum proteolysis (protease molecules vastly outnumber the inhibitor molecules in the immediate pericellular zones)^34^ and in part due to the fact that liberated proteases can bind to the neutrophil cell surface, rendering them resistant to protease inhibitors by steric hindrance.^35^ Our experimental findings suggest that hypoxia will augment these processes to promote adjacent tissue injury.

Histotoxic proteases such as NE, MPO and MMP-9 are present in COPD sputum and bronchoalveolar lavage fluid, and the levels correlate with the indices of disease severity.^24^ Likewise, in the context of evidence for airway hypoxia in CF, both NE and MMP-9 are linked to the pathogenesis and progression of this condition.^23^ We therefore propose that tissue hypoxia engenders a detrimental neutrophil phenotype, with enhanced injurious potential but diminished bactericidal capacity;^22^ furthermore, hypoxic prolongation of neutrophil lifespan will increase tissue exposure to these damaging effector cells. This process may also contribute to the pathogenesis of a range of other tissue-destructive conditions where hypoxia is relevant, for example, granulomatous inflammation,^36^ pleural sepsis and abscess formation. These scenarios underscore the need to study neutrophils under conditions of hypoxia and to determine the mechanism(s) that lead to this aberrant functional phenotype.

HIFs are the key regulators implementing hypoxic cellular responses, but although we identified putative hypoxia response elements (HREs) in the promoter sites of MMP-9 and NE, there was no increase in their mRNA transcript or protein levels in hypoxic neutrophils. Together with the failure of hypoxia mimetics to recapitulate, and of cycloheximide to suppress hypoxia-augmented degranulation, these data suggest a HIF-independent mechanism. Since calcium flux in neuronal cells has been reported to be increased by hypoxia,^37^ we investigated the impact of modulating intracellular calcium levels on degranulation under normoxic and hypoxic incubation. Thapsigargin increased NE release, compatible with the presumption that neutrophil degranulation is a calcium-dependent function. Although EGTA reduced degranulation, a substantial release of NE was still detected under hypoxic conditions. This is consistent with a recent report that STIM1-deficient murine neutrophils show loss of store-operated calcium entry but only a minor defect in fMLP-induced degranulation (although lactoferrin alone was assayed in this study).^38^ The release of calcium from intracellular stores will be preserved in the presence of EGTA, and it is possible that this is sufficient to initiate some degranulation responses.

PI3Kγ is a key mediator of neutrophil degranulation, and has been reported to be upregulated by hypoxia,^39^ as has activation of the effector kinase AKT.^40^ We found that treatment with the pan-PI3K inhibitor LY294002 abolished the hypoxic uplift of neutrophil degranulation, an effect that could be fully recapitulated by the isoform-selective PI3Kγ inhibitor AS605240 but not by IC87114 (a compound with relative selectivity for PI3Kδ). In keeping with a role for the PI3K pathway, hypoxia upregulated the phosphorylation of AKT in response to GM-CSF; however there was no effect on the subsequent response to fMLP, suggesting that the early increase in PI3K-dependent signalling entrains other (as yet unknown) cellular responses or pathways. Surprisingly, given that PI3Kγ is generally coupled to ligation of G-protein, rather than tyrosine kinase-linked receptors, inhibition of PI3Kγ abolished the AKT phosphorylation in GM-CSF-treated neutrophils. How hypoxia might modulate PI3Kγ activity independent of HIF is currently unknown and is a focus of ongoing investigation in our laboratory.

In summary, levels of hypoxia encountered by inflammatory cells in pathophysiological situations increase neutrophil degranulation, deploying harmful proteins and proteases to the extracellular milieu and increasing the capacity for tissue injury. The mechanism by which hypoxia engenders this hyperactivated state does not relate to the established HIF-dependent signalling pathways, but may involve PI3Kγ.
