# Supplementary material for: Hypoxia upregulates neutrophil degranulation and potential for tissue injury
Source: Thorax. 2016 Aug 31;71(11):1030–8. doi: 10.1136/thoraxjnl-2015-207604 (PMC5099189; doi:10.1136/thoraxjnl-2015-207604)

Supplemental Figure 1: Increased granule release is not secondary to apoptosis, necrosis or NET formation.

A. The pH of the media was measured under normoxic and hypoxic conditions, with no significant differences recorded throughout the incubation period. B&C. There is no increase in apoptosis under the degranulation assay conditions. Neutrophils were incubated under normoxia (N) or hypoxia (H) for 4 h prior to treatment with GM-CSF (10ng/ml, 30 min) and fMLP (100nM, 10 min) (GM) or media control and stained with May-Grünwald-Giemsa (B). C. Neutrophils were incubated under normoxia (N) or hypoxia (H) for 4 h prior to staining with FITC annexin-TO-PRO-3 (Invitrogen). Healthy cells, early apoptosis, late apoptosis and necrosis are represented by double negative, Annexin positive, double positive and TO-PRO-3 positive respectively. D. NET formation is not increased by hypoxia. Neutrophils were incubated under normoxia (N) or hypoxia (H) for 4 h prior to treatment with GM-CSF (10ng/ml, 30 min) and fMLP (100nM, 10 min) (GM), PMA (20mM) or media control. NETs were quantified with cell-impermeable nucleic acid dye Sytox Green (1:1000) in a fluorescence microplate reader at baseline, post 4 hour incubation and post treatment. NETs are expressed as extracellular DNA as % of total DNA from triton-X (0.5%) lysed neutrophils.

Supplemental Figure 2: CSM does not affect degranulation

A: Neutrophils were incubated under normoxia or hypoxia ± CSM (25-50%) for 4 hours prior to priming with GM-CSF (10ng/ml, 5 min) and activation with fMLP (100 nM, 10 min). Degranulation was measured by elastase activity assay. Results represent mean ± SEM (n=3) B: CSM induced intracellular stress. Neutrophils were loaded with DC-FDA (0.2μM) for 10 min prior to exposure to a range of CSM concentrations (5-100% CSM, 30 min) or PMA (200nM, 10 min). The production of intracellular ROS was analysed by flow cytometry (h). Results represent mean ± SD (n=2, each performed in triplicate)

Supplemental Figure 3: Neutrophil supernatant-mediated damage to primary lung epithelial cells

A. Primary ciliated HBECs were scraped from a Transwell® insert and exposed to normoxic and hypoxic neutrophil supernatants (1 in 2.5 dilution) for 2 h. Cells were fixed with Sorensen phosphate-buffered (pH 7.4) glutaraldehyde containing 0.1% (v/v) hydrogen peroxide and osmium tetroxide (2 h at 4^0 C), embedded in Spurr's resin and imaged using a Philips CM 100 TEM. Representative images of a single experiment, 6 images per treatment, magnification = x2500. B. ALI cultures of primary HBECs were exposed to neutrophil supernatants (1:2.5 dilution, 500 µl) applied to the basal surface. 10µl samples were aspirated from the basal well at 0, 4, 8, and 24 h and analyzed by commercial LDH assay (Sigma). Results represent a single experimental procedure with all conditions run in duplicate.

Supplemental Figure 4: AKT phosphorylation of serine 473 under hypoxic conditions

Hypoxic AKT serine-473 phosphorylation is increased after 15 min of GM-CSF stimulation and this is PI3K-γ dependent. Neutrophils were incubated under normoxia or hypoxia for 4 hours prior to priming with GM-CSF (10ng/ml, 15 min or 30 min). C&D AKT phosphorylation is further increased by fMLP stimulation. Neutrophils were incubated under normoxia or hypoxia for 4 hours prior to priming with GM-CSF (10 ng/ml, 15 min or 30 min), prior to activation by fMLP (100nM, 10 min). p-AKT was quantified by western blotting. Results represent mean ± SD.

Supplemental Movies: granule products affect ciliary function

Primary HBECs grown in ALI culture were scraped from the Transwell® inserts, transferred to a chamber slide and exposed to undiluted neutrophil supernatants for 2 h. Recordings

were made at 0 and 2 h with a high-speed camera (500 fps). Ciliary beat frequency determined by analysis of n=3 movies with CiliaFA at t=2 hours; fresh cells 0 hours.

S. Figure 1
Hoenderdos et al.

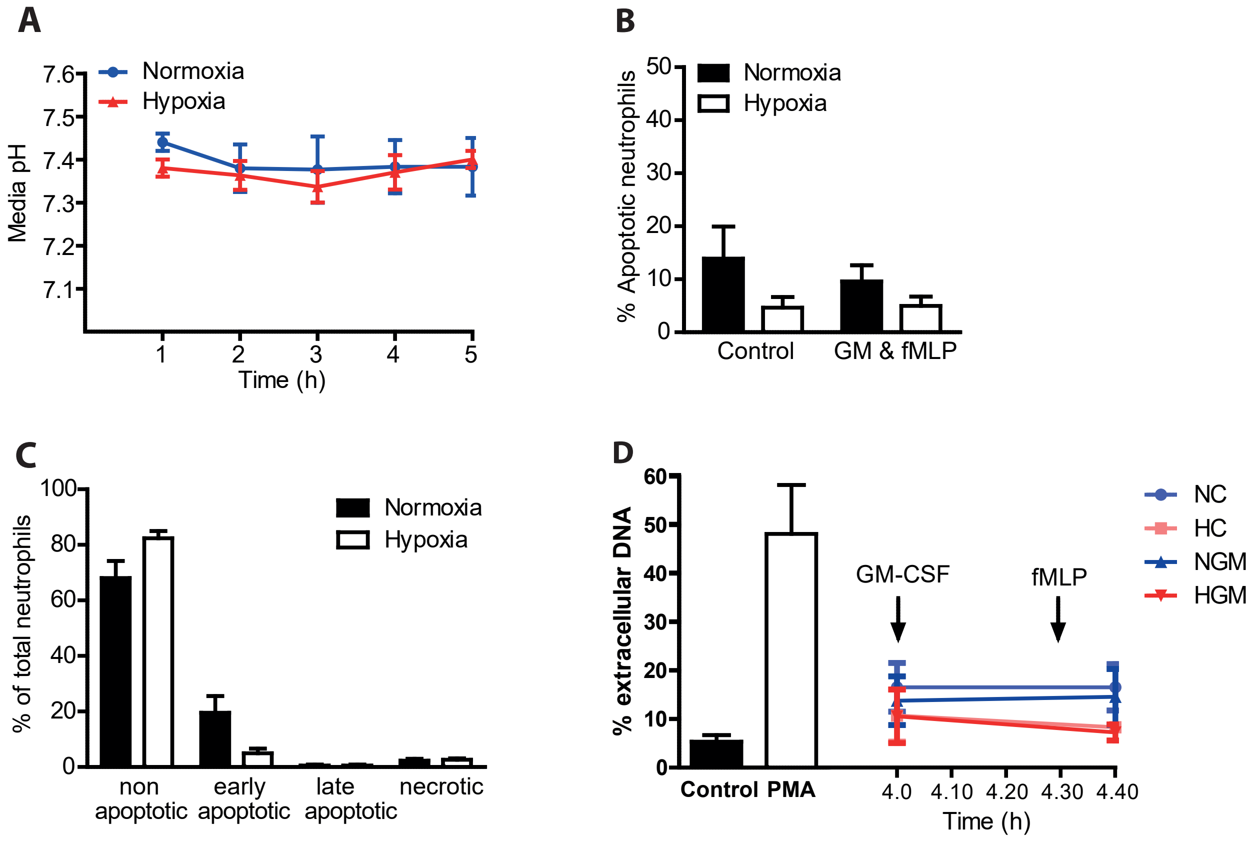

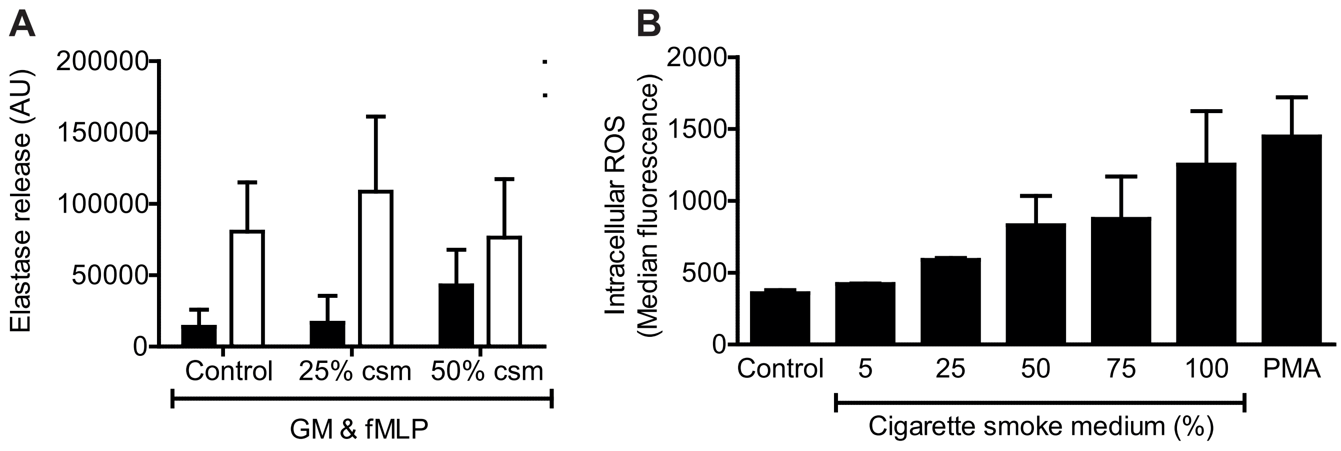
A
Elastase release (AU)
200000
150000
100000
50000
0
Control
25% csm
50% csm
GM & fMLP
B
Intracellular ROS
(Median fluorescence)
2000
1500
1000
500
0
Control
5
25
50
75
100
PMA
Cigarette smoke medium (%)

S. Figure 3
Hoenderdos et al.

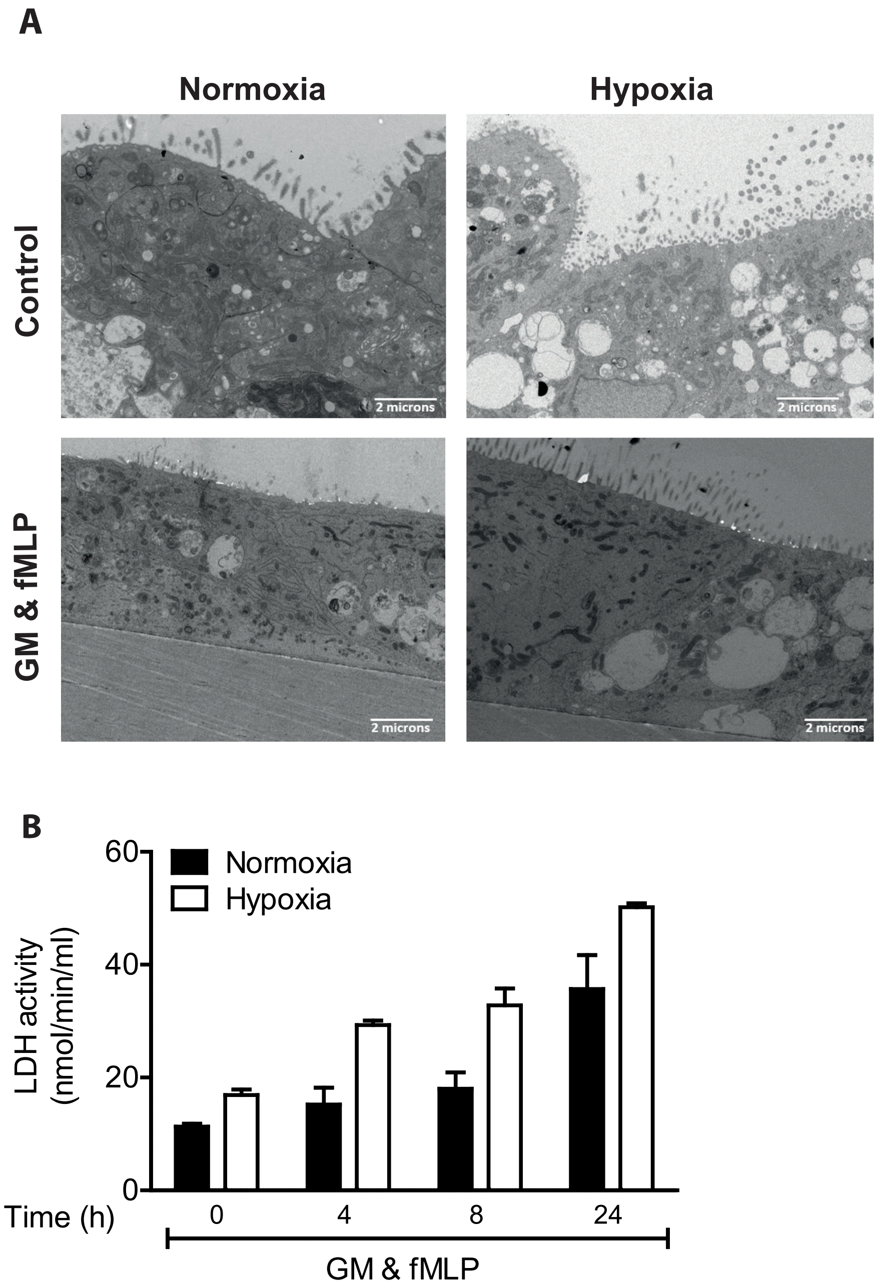

S. Figure 4
Hoenderdos et al.

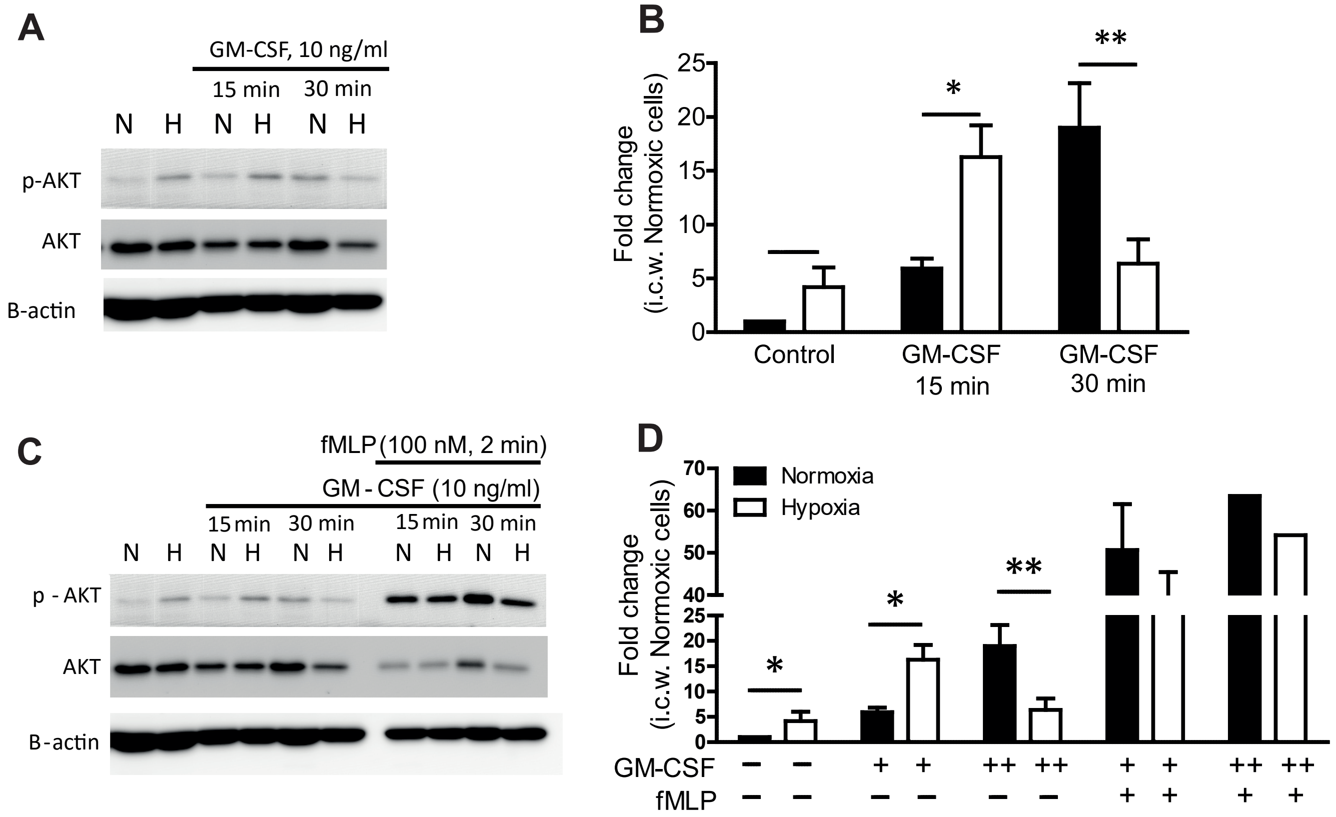

Supplement: supplementary figures [file thoraxjnl-2015-207604supp001.pdf]
